# Supplementary material for: Radiopharmaceutical Labelling for Lung Ventilation/Perfusion PET/CT Imaging: A Review of Production and Optimization Processes for Clinical Use
Source: Pharmaceuticals (Basel). 2022 Apr 22;15(5):518. doi: 10.3390/ph15050518 (PMC9143102; doi:10.3390/ph15050518)
Supplement: Supplementary file 1 [file pharmaceuticals-15-00518-s001.zip › pharmaceuticals-1661929-supplementary.pdf]

Table S1. [^{68}Ga]Ga-MAA particles size and biodistribution study according to the various authors.

	Particles mean Ø (µm)	Biodistirbution study		*In vitro* stability	
		Animals		Animal serum (37°C)	Human serum or plasma (37°C)
		[^{68}Ga]Ga-MAA tested in animals	Activity injected (MBq)		
Even and Green [24]	10.0 - 90.0	no			
Maus *et al.* [32]	3.0 - 150.0 (3.0 - 40.0 for 95.0%)	Sprague Dawley rats (n=2)	10.3 ± 1.5		
Ament *et al.* [33]	3.0 - 150.0 (3.0 - 40.0 for 95.0%)	Sprague Dawley rats (n=2)	10.3 ± 1.5		1h (plasma)
Amor-Coarasa *et al.* [34]		Sprague Dawley rats (2/T°C)	1.8 à 3.7		
Jain *et al.* [29]	52.9 ± 15.2	Normal swiss mice	0.4	45 min	
Shannehsazzadeh *et al.* [36]	24.31	normal female wild-type rats	6.6 (static PET/CT images) 1.8 MBq (dynamic PET/CT images)		
Persico *et al.* [37]	7.0 - 100.0	no			1h
Blanc-Béguin *et al.* [40]	15.0 - 75.0 (25.0 - 50.0 for 70.0 %)	no			
Canziani *et al.* [18]	43.0 - 51.0	no			
